# A feminist approach to eating disorders in China: a qualitative study

**DOI:** 10.1186/s40337-023-00883-z

**Published:** 2023-09-14

**Authors:** Su Holmes, Hua Ma

**Affiliations:** Department of Film, TV and Media, University of East Anglia, Norwich, NR4 7TJ UK

**Keywords:** China, Eating disorders, Feminism, Transcultural, Bulimia, Binge eating disorder

## Abstract

**Background:**

As women continue to be more at risk from eating disorders, gender has often been a focus of concern in transcultural research. Yet feminist, qualitative studies which prioritize the voices of women/girls remain rare within transcultural work suggesting the need for greater interaction between these fields. This article seeks to contribute to the exploration of the applicability of feminist paradigmslargely developed in the West—to experiences of EDs in non-western contexts.

**Methods:**

This article draws on semi-structured interviews with 12 women from urban China with self-reported experience of Bulimia Nervosa (BN) and binge eating disorder (BED) in order to explore the complex ways in which gender may be implicated within eating/body distress from a transcultural point of view. The data is analysed through Reflexive Thematic Analysis.

**Results:**

The data analysis suggested two broad themes: (1) Chinese versus Western codes for judging female appearance: from surveillance to liberation (2) Discipline, appetite and control: the gendered/cultural meanings of binging and purging. In terms of the first theme, many participants had spent time in the West which was understood as a less regulated context in terms of gendered body surveillance and eating. Complicating existing assumptions about the ‘Westernisation’ thesis, different communication codes and peer interactions across Chinese and Western contexts played a central role in how participants experienced their bodies. In the second theme, binging and purging emerged as a way to manage a number of contradictions surrounding Chinese femininity, including respecting familial food cultures, contradictory discourses on female ‘appetite’, and the need to display a female body which signified cultural imperatives of self-restraint and discipline.

**Conclusions:**

The data emphasises the importance of examining the culturally specific meanings of eating problems and their gendered contexts, whilst there is clearly much that echoes Western feminist work on Western samples. Although limited, the study crucially points to the importance of examining how ED subcategories other than AN can be explored from a transcultural and feminist point of view.

## Abstract (plain language summary)

This articles adopts a feminist approach to explore experiences of eating disorders (EDs) in China. Drawing upon interviews with 12 women from urban China with experience of self-reported Bulimia Nervosa (BN) and Binge Eating Disorder (BED), it examines the relationships between eating problems and gender—from social norms regarding body and appearance to women’s social roles. The vast majority of feminist work on EDs has been developed in the West. The article explores how applicable this research is to these women’s experiences in China, whilst examining how the Chinese context highlights culturally specific aspects of EDs.

## Introduction

Although once largely seen as the province of Caucasian, cis-gender women in the West, eating disorders (EDs) are now understood to be a global problem. In China, the prevalence of EDs has been increasing since 1990 [1, 2]. The prevalence of disordered eating could be as high as 7.04% in the general Chinese population [3] and levels may equal or exceed those in Western contexts such as the US [4]. As in the West, boys and men are increasingly affected by body dissatisfaction and eating problems [2] and—given the significant correlation between EDs and gender minorities in the West—work is beginning to address the relationship between eating pathology in Chinese transgender/non-binary populations [5]. But cisgender women remain most at risk from EDs in China [2]. Age also remains a key predictive factor: adolescents are the most likely to be affected followed by young adults [2]. Although epidemiological studies are limited, research points to Bulimia Nervosa (BN) and binge-eating disorder (BED) as being more prevalent than Anorexia Nervosa (AN) [4]-broadly mirroring statistical evidence from the West [6].

In transcultural work on EDs, focus was initially given to the concepts of Westernization and acculturation [7–9]. But research has increasingly demonstrated the need to examine how EDs in non-Western contexts are ‘aetiologically and phenomenologically distinct from the eating disorders named, diagnosed and treated in the West’ [10]; the nuanced ways in which EDs are ‘culturally reactive’ but not necessarily ‘culture-bound’ [11] (interacting in complex ways with their cultural contexts but not restricted to particular contexts) and how processes of Westernization work alongside nationally-specific cultural mores and transformations [11]. In this regard, given the extent to which gender is key risk factor for the development of EDs, it has been a focus in transcultural research (e.g. [7, 12, 13]). Yet in transcultural work, feminist, qualitative studies which prioritize the voices of women/girls remain rare. In a landmark article in 1997, Katzman and Lee asserted the need to bring feminist approaches into clearer dialogue with transcultural studies of eating problems. Whilst feminist approaches historically focused on white, Western, middle-class women, earlier transcultural work prioritized Westernization (rather than gender) as the organizing frame [9]. Whether conceptually or empirically, there have since been efforts to address this gap [14–16]. But Pudge was still able to comment in 2022 that the conclusions drawn by transcultural and feminist scholars ‘reflect the conceptual bounds of their disciplines by treating the localised and gendered aspects of culture as mutually exclusive categories of experience’ [10].

In responding to this context, the current article draws on interviews with 12 women from urban China (most with self-reported BN/BED) in order to explore the ways in which gender is implicated within eating/body distress from a transcultural point of view. In particular, it seeks to contribute to the exploration of the applicability of feminist paradigms—largely developed in the West—to experiences of EDs in non-western contexts [14, 15]. It is fully acknowledged that Western feminism has historically been critiqued for its ethnocentrism [17]. In this regard, always adopting it as the default ‘starting point’ is itself problematic as such an approach enables Western feminism to set the terms of analysis. Yet as feminist scholarship on EDs originates from the West, it offers a logical and necessary starting point here. We seek to explore the relevance of Western feminist approaches to EDs in China whilst highlighting how non-Western voices and experiences enable an understanding of the gaps, omissions and limitations in this field.

## Research background

### Western feminist approaches to eating disorders

Western feminist approaches to EDs have something of a 45-year history and emerged out of the second wave’s focus on the ‘personal as political’. Pioneering scholars in the field situated the apparent rise in eating problems in relation to the consequences of the Women’s Movement, and the resulting contradictions and pressures surrounding female roles [18, 19]. With a primary focus on anorexia (AN), eating problems were situated in a context in which dieting and calorimetry were a normal preoccupation for women and were understood as existing on a continuum with normative femininity [20–23]. But EDs were not simply figured as an indication of patriarchal backlash, and there has long since been an emphasis on the shifting and contradictory meanings embedded within ED bodies, practices and selves [24]. Largely conceptual or qualitative, feminist work has emphasised how eating/body distress may be related to objectification and gendered embodiment [25, 26] sexual harassment; rape/sexual abuse; a desire to evade or ‘opt out’ of gender binaries and sexual availability [20, 21, 27]; an attempt to stall transition into a heavily gendered culture in which women may *not* be able to ‘have it all’ [28]; constructions of female ‘appetite’ (sex/food/career) [29]; the overvaluation of women as nurturers; and conflicting discourses of consumerism, globalization and healthism [22, 27, 30, 31]. In comparison to sociocultural approaches which focus more on the relationship between EDs, gender and body dissatisfaction [32], feminist approaches have often explored the relationship between broader changes in the construction of women’s social roles and the implications for these for eating/body distress [33]. In much feminist work it is understood that efforts to conform to/and/or resist discourses of ‘restricted agency’ [26] may find expression *through* embodiment, rather than necessarily taking body dissatisfaction or body image concerns as their central core [29, 34].

There have been efforts to examine the intersectional impacts of misogyny, racism, poverty and heterosexism in Western feminist work on eating problems [16, 35, 36], and some of this was work there from an early stage [37]. But a focus on white, Western cisgender girls/women with AN has prevailed (and little feminist work has analyzed the qualitative experiences of males, trans and non-binary individuals diagnosed with EDs). Feminist work has thus been accused of ‘overvaluing sexism’ at the expense of other intersectional factors [9]—critiques which mirror wider shifts and tensions within feminism(s) more broadly. Transcultural perspectives thus continue to be marginalized within feminist scholarship, limiting the extent to which culturally specific meanings of eating, food refusal and bodies and agency [16] can be understood in relation to structural gender inequalities and experiences.

In addition, the majority of feminist, as well as transcultural work on EDs has focused on AN, in part because the ‘anorexic’ body is seen as remarkable or extraordinary—apparently offering a more arresting visual example of gendered inscription [29, 38]. As Squire observes, the ‘bulimic’ body offers a ‘more ambiguous site’ which is less amenable to ‘visually based interpretations’—one so not easily adopted as an emblem of gender oppression [38]. In this regard, although similar gendered contexts and inequalities may give rise to these different presentations of eating/body distress, their *cultural meanings* may diverge. Despite this, there is little work that has explored these different experiences within non-Western countries. This is a considerable omission in both transcultural and feminist research on EDs which contributes to the hierarchical visibility of ED subtypes and the silencing of a broader range of ED experiences in different cultural contexts.

### Gender and beauty ideals in China

The feminist emphasis on how conflicting feminine ideals in society and rapid social change foster the emergence of EDs [9, 19, 33], is particularly pertinent to the Chinese context. The influence of traditional ideas of Confucianism on the status and roles of women in China has been well researched [39, 40]. Confucianism endorsed male superiority and emphasized women’s submission and subordination: women and men were assigned clear roles in the family and women had little power outside of the home [41]. After new China was built in 1949, the Chinese Communist Party promoted a contrasting ideology of gender equality through political and media discourse (a popular slogan was ‘Women can hold up half sky’ [42]). Furthermore, women and men were encouraged to wear unisex clothes which reflected the emphasis on gender neutrality within Maoist ideology, and the Communist Party encouraged women to abolish beauty practices to achieve women’s liberation as one of their revolutionary goals. This abolition was believed to free women from objectification and introduce greater social equality [43]. However, the Maoist conception of gender equality was dismissed during the economic reform in 1979, and during the post-Mao period, the biological notion of gender differences was re-emphasized [44].

The development of the beauty industry as part of the post-socialist economy then had a significant impact on the construction of gender ideals. Indeed, people refer to contemporary China as ‘the era of face-judging’ (kàn liǎn shí dài, 看脸时代)—a discourse which has unequal consequences for women [45]. Young Chinese women are encouraged to value and pursue strict requirements for female beauty [46]. These include normalized beauty standards such as ﻿as ‘a watermelon seed shaped face; a sharp chin; a tiny face; big eyes; double eyelids; a tall nose; and [the] physical features … [of] being tall and slim’ [47]. Similarly, bái shòu yòu (白瘦幼) translates as ‘white, slim and young’ and is a common/popular expression that embodies aspects of typical, female beauty standards in China.

The extent to which young women in China recognise the currency of thinness to social acceptance, relationship and employment success has been explained in relation to the impact of Western beauty standards [15, 41, 48]. It has been suggested that East Asian culture historically associated ‘slenderness among women with poverty, poor health, and low fecundity’ [33, 49]—marking it out as undesirable. Yet such meanings have been subject to change. Jung’s study of how young Chinese women perceive traditional and contemporary beauty ideals revealed that in addition to a ‘round face’, ‘small lips’, ‘small eyes’, ‘long black hair’ and ‘small feet’, traditional ideals were seen as endorsing ‘fat’ or heavier female bodies compared to current aspirations for thinness [41]. At the same time, there has been little empirical work which has investigated women’s *perceptions* of these (‘Western’) influences, and metanarratives may assume a problematic dynamic in which women ‘passively absorb… Western templates of beauty’ [11].

As noted, China was traditionally shaped by the gendered values of Confucian philosophy which emphasised women’s submissiveness to men and primary association with the domestic sphere, as well as filial piety and ‘community-based individuality’ [15]. Yet the transformation of Chinese society to a market economy significantly impacted social roles for women. Traditional expectations of gender and family structures were called into question by the rise in women taking up work and educational opportunities, even though gender inequalities remain starkly apparent [11]. Following wider feminist arguments about the relationship between social change and eating problems [19, 33], this conflict has been ventured as a context for eating problems in China. As de Montgrémier et al. explain, the ‘tension between the so-called “international” modern, liberal, and individualist Western culture and the traditional collectivist Chinese culture engenders a form of internally … contradictory discourse that might make young women vulnerable and susceptible’ to eating problems [15]. de Montgrémier et al.’s study of eleven adolescent girls/young women hospitalized for an ED (and eight of the participant parents) represents one of the only feminist qualitative studies of EDs in China. On one level, the authors found similar trigger factors for EDs to those in Western contexts, such as educational and work pressures; the social currency of thinness, and a desire for independence, autonomy and control [15]. At the same time, although patriarchal control and the oppression of women may foster similar methods to achieve feminine agency and autonomy across cultures [9, 33], the authors’ understanding of the specific gendered context here suggests that the ‘will to be thin reflects a meaning different from that in Western countries’ [15]. In this respect, their study offers a useful foundation for approaching the interplay between gender, culture and EDs in the Chinese context. But despite having participants with a range of ED diagnoses—including AN; AN binge purge type; BN—these differences are not discussed at all and an emphasis on thinness (as the quote above suggests) prevails. Qualitative studies are of course also limited in their generalizability, and different samples can yield very different results.

This summary of the existing relations between feminist and transcultural work on EDs suggests a number of omissions, trends and tensions that we seek to address and explore in this article. First, there has been little systemic, epistemological or methodological discussion of the relations between feminist and transcultural approaches to EDs since Katzman and Sing launched the dialogue in 1997—limiting our understanding of the opportunities and/or barriers for cross-fusion. Second, feminist qualitative research which prioritizes the voices of those with experience of an eating problem has played a more marginal role in shaping transcultural understandings of EDs than we might expect (given the emphasis on gender in transcultural research in the field). Yet such methods can offer an important challenge to quantitative and/or biomedical approaches to EDs and enable an important means of understanding of how eating/body distress are *experienced* in culturally-specific ways. Third, both feminist and transcultural research has marginalised the experience of eating problems outside of AN, enabling an emphasis on the pursuit and meanings of thinness to dominate. This reduplicates an existing scholarly and cultural bias in the West—contributing to the silencing of a wider range of ED experiences and the ‘double pathologization’ [29] of women who binge/purge. Although the limited data we shall discuss for the present study in no way claims to ‘solve’ or overcome these challenges and omissions, it seeks to reinvigorate debate about the relationships between feminist and transcultural approaches to EDs by using the insights from a rich culturally-specific sample.

## Methodology

### Recruitment

After ethical approval was received by the authors’ institution in February 2023, participants were recruited and interviewed via the social media platform (SMP) WeChat. Using snowball sampling, the study began with recruitment posts on one of the researcher's Chinese SMPs including Little Red Book, Douban and Wechat. People were eligible for the study if they were Chinese (both parents being Chinese citizens, born and grown up in mainland China, and native language is Chinese); identified as a woman and understood themselves to have experience of an ED. The study recruited 12 participants who came from different provinces in China (predominantly urban). All had experience of a self-reported ED and most suggested that they were now recovered from their eating problem. Six participants described themselves as having BN, four as BED and two did not feel certain about categorizing their problem. Ages ranged from 20 to 29 with a mean age of 25. Participants identified as pansexual (n = 1), lesbian (n = 2), bisexual (n = 3) and heterosexual (n = 6). At the time of the study, most of the participants had spent the majority of their lives in China. They all resided in China until the age of eighteen and over half of the participants had been or were engaged in overseas studies in the US/UK. Indeed, all of the participants were educated women: they were either university graduates or currently studying for a higher degree. Many had also travelled abroad (US/UK) to study in ways which emerged as significant to the data in terms of comparisons between Chinese and Western cultures.

Given the difficulty of being openly gay/bisexual in the Chinese context [50], the demographic recruited for the study was something of a surprise to the researchers. It could be that snowballing encouraged people within similar networks to respond. In addition, the research attracted a number of university educated students who were studying gender—a pursuit which may be more associated with marginalized communities in the Chinese context. Western studies have explored how occupying a sexual minority identity may increase the risk for EDs (although the results are uneven and there is more work on males) [51], and how experiences of sexual marginalization can shape the political and personal meanings of eating/body distress [36]. Nevertheless, the participants in the study who identified as pan/bi/lesbian did not refer to issues of sexuality in interview, whether explicitly or implicitly. The focus of discussion for all the participants was the role women and women’s bodies within a heterosexual economy, perhaps suggesting the dominance of this framework to their lived experiences.

In terms of eating pathology, most participants discussed experiences of restriction, binging and purging, although self-starvation was more marginal here. The particular emphasis on *compensatory* behaviours—especially binging/purging—are more characteristic of Western understandings of BN than BED (which typically involves no such compensatory strategies) [52]. But as Sun et al. observe in their study of popular discourse on EDs in China, the terms ‘BN’ and ‘BED’ can be used interchangeably, so it is crucial to acknowledge cultural specificity here [53]. Indeed, given the fact that existing diagnostic discourses assert a Western hegemony [8], as well as the fact that feminist approaches critique medical constructions of EDs more widely [20–22], the researchers accepted the terms (or lack of them) used by the participants and simply note that cross-cultural differences are at work here. Furthermore, the fact that the study recruited participants who had *not* received a clinical diagnosis may reflect differing approaches to conceptualizing EDs in the Chinese context. Existing research suggests that symptoms other than those related to EDs may often be prioritised [1]. Some of the participants in this study were diagnosed with and treated for depression or anxiety, despite presenting with marked physical and psychological symptoms of an eating problem. There is also less specialist treatment available in China and stigma may be an even greater barrier to help-seeking than in the West [54, 55]. These factors then likely further impact the number of people that receive an ‘official’ diagnosis.

### Procedure

As the second-named author is Chinese and bilingual she organized, conducted, transcribed and translated the interviews. Consent was given by participants via email prior to interview (using a consent form). Seven of the interviews were conducted via video call on WeChat; four were conducted via WeChat using audio alone, and one interview was conducted face-to-face. Each interview lasted for 60 min on average. The interview schedule covered several overlapping themes including participant views on what they understood an ‘ED’ to be; why they think developed an ED; whether they perceived a relationship between cultural constructs of gender and eating problems (and if so, why how); whether and/or why they saw EDs as a gendered issue. They were offered a de-briefing sheet after the interview and were given the opportunity to check or comment on their transcript. Participants chose their own pseudonyms for anonymization purposes.

Both authors are women with personal experience of an ED and they chose to briefly disclose this on the participant information sheet and within the interview context itself. The discussion of personal experience and reflexivity was in part prompted by the long-running debate about the interviewer–interviewee relationship in feminist work and the power dynamics within which this takes place [56, 57]. As a woman born and raised in China, the interviewer also had first-hand experience of the culture the participants were discussing which could work to facilitate a rapport. Yet the possibility of a ‘non-hierarchical’ [56] relationship based on gender congruence and shared cultural experience has long been subject to critique. Feminist work has explored the range of intersectional factors which shape the balance of power within the interview encounter [57, 58], whilst also arguing that this is context-specific and shifting. For example, whilst it is important to recognise ‘the power structures within academia itself’ [58], the educational levels of the participants complicated any neat binary here between participant/interviewer, and they were often highly literate in approaching the issues in hand (see below).

### Analysis

The interview data was approached using reflexive thematic analysis (RTA) [59]—a theoretically flexible approach to qualitative data that enables the identification of themes or patterns [60]. RTA is ‘collaborative and reflexive, aiming to achieve richer interpretations of meaning, rather than attempting to achieve consensus of meaning’ [60]. Indeed, RTA suggests that meaning emerges from the intersections between the dataset; the conceptual/theoretical assumptions of the analytic framework; and the perspectives/subjectivities of the researchers [60]. This processes of negotiation and exchange—and the acknowledgement of researcher subjectivity—was particularly pertinent here given the different national and ethnic identities of the researchers: one is a Chinese woman and the other a white Western woman. Whilst the first author brought particular experience of feminist approaches to EDs, the second author’s familiarity with Chinese values and culture enabled the interviews to be transcribed and analysed in a culturally-sensitive manner. That is, she would also help to ‘decode’ cultural meanings that may be more unfamiliar to a Western gaze (and thus the understanding of the other author). This included, for example, understanding the apparent ‘directness’ of Chinese peer/family communication discussed in the analysis of the first theme below.

In line with an RTA approach, the first stage involved familiarization with the data; so the transcripts were read and re-read by the researchers separately so as to produce notes on preliminary ideas and observations. This was not done to ‘test’ the reliability of the process but—particularly given the different backgrounds of the researchers—to try and enable multiple (and rich) interpretations of the data [59, 60]. Second, this process was used to generate initial codes across the data set (and codes here are understood as individual data items which are the basic building blocks for later themes) [60]. Further familiarization with the data and the development of codes work together [59], and initial codes listed by the authors were: gender inequalities/expectations in China; the prevalence of peer comments on body/eating; EDs as stigmatized/silenced in China; EDs as a ‘women’s’ problem that did not really effect men; peer discourse on the body in Western contexts. In third stage, the codes were used to begin to explore broader themes and connections across the dataset. In RTA, themes do not reside in the data ready to be ‘located’ by the researcher, but are rather constructed via the researcher(s) actively construing the relationships between their version of the codes [60]. This stage can lead to the combining, discarding or renaming of particular codes, or finding contradiction or complexity within them [60]. The key themes arrived it in stage 3 were (1) Chinese versus Western codes for judging female appearance: from surveillance to liberation (2) Discipline, appetite and control: the gendered/cultural meanings of binging and purging. In step four these themes were reviewed (to reflect, for example, on the boundaries of the theme; its coherence and its importance in relation to the purpose of the research) [60]. In step five, the thematic categories were analyzed in detail and data extracts that best represented these themes were selected for inclusion. In stage six, the writing then involved placing the themes in relation to Western feminist paradigms for understanding EDs, as well as contextually specific literature on gender in the Chinese context.

## Results and discussion

### Theme #1: Chinese versus Western codes for judging female appearance: from surveillance to liberation

#### Experiences of surveillance/self-surveillance in China

Scholars have long since acknowledged the contributions of the Eurocentric thin ideal and acculturation pressures to the development of eating and body-related distress in Asian women [33, 61]. Attention has been given to Western influence coming *into* a host culture [61] as well as the impact of acculturation on women from other cultures living *in* the West [7, 14] As discussed, a number of the participants in the sample had studied in the Western contexts (UK/UK), enabling a consideration of cross-cultural experiences here. We first consider the participants’ understandings of body regulation and peer comments in their native China, before examining what emerged as contrasting discussions of their overseas experiences.

Existing sociocultural work has foregrounded the central role played by peer/familial comments on the body in experiences of eating problems/body dissatisfaction in China [62, 63]. These findings were also supported by the current study. Participants offered recollections of external comments and judgements on their bodies and appearances from a young age, and these experiences extended into adulthood. Such discourses were seen as everyday, expected and pervasive, but also wounding and painful, with moments or comments often emerging as vivid memories in the narrating of food/body distress. Participant Bagel relayed a conversation with a female peer in which she was told ‘If I saw you looking like your [passport] photo [when you were slimmer], I would really like you’, and others recalled similar comments from educators or family members. But although this was seen as expected and normative, the participants were often critical of what they saw as the sexist nature of such structures, and persistently called out the gender discrepancies in body/appearance shaming. As ChengCheng explained: ‘I didn’t know before, but now I know that being a teenage girl in this society, there is no way to escape being shamed [for your body and appearance]’. Julia suggested that:

So I feel that boys don’t have this kind of appearance anxiety. Even if a boy feels that he’s 250 or 260 pounds… not many people will ask him to lose weight or anything, and he himself won’t have that kind of consciousness that he must lose weight.

According to the women, such gender disparities were a key reason why EDs were predominantly associated with women. Such power dynamics were understood as a form of external regulation—from boys/men, female peers, parents and educators—which was then internalized through self-surveillance and self-objectification [20]. As Xiaoyang observed: ‘This …is the feeling of social surveillance. Eventually, it will become self-surveillance and self-censorship’.

As this comment suggests, and likely because some were familiar with critical frameworks surrounding gender from their studies, the participants were keen to discuss EDs within a feminist framework, and it was not a case of the study somehow ‘enlightening’ them in this regard. It is equally worth noting that whilst they talked about the social currency of thinness for women, they also focused on the interrelations between the regulation of female bodies and the construction women’s *wider* social roles—adding credence to the feminist argument that these are intimately intertwined. So as ChengCheng observed:

Body shaming is disciplining you, making you manage your body… [M]ore bluntly … it’s a patriarchal society, hoping that you can manage your body well and become a perfect new era woman, to work well, to be a good wife and mother, and to become the ideal image of a woman that society expects (ChengCheng).

In objectification theory, women ‘assimilate to an observer-oriented self-perception not out of self-absorption, but as an anticipatory strategy that allows for a modicum of agency in how one will be regarded by others’ [64]. As with Xiaoyang’s comment above, ChengCheng slips between a focus on external regulation (‘making you’) and internalisation of this framework (in which ‘hoping that you can… become the ideal’ appears to be the only ‘agency’ on offer).

Participants spoke of the micro-surveillance of their bodies in the context of peer and family comments. Numerical measures of weight were commonplace within everyday body talk, and these intersected with wider regulatory discourses on female appearance:

Although [my mother]… is not very thin, she still insists that I cannot weigh more than 100 pounds, and even now, when I go to work without makeup, she thinks I look sloppy, and she thinks I should always be fully made up, and she’s someone who has very strict requirements for appearance (Sihui).

In de Montgrémier et al.’s [15] study of young women and EDs in China the data emphasized generational conflict: parents did not really understand the currency or pursuit of thin ideal which was seen as the result of Western influence. Yet in the current study this was rarely the case, and mothers in particular emerged as key mediators of corporeal/beauty norms. Nor was this always presented as a ‘new’ concern: as Xiaoyang said of her mother, ‘I think she’s *pretty traditional* and feels that being overweight isn’t very attractive [our emphasis]’, suggesting that it is not clear-cut to distinguish between the modernising influence of western ideals and a culture in which has long since valued thinness in women [11].

In terms of contextualizing the central role of peer comments in the data, it has been suggested that the influence of peer and family comments is heightened in collectivist societies such as China. There is a stronger normalization of social conformity and thus a keener pursuit of prevailing social standards [63]. But the stories from the participants also highlight cultural specificities of communication here. So what may appear to be the explicit and direct nature of the comments can be contextualized in relation to particular modalities and scripts of Chinese communication [65]. Chinese communication is organised around a distinction between insider-communication (zijiren, 自己人) (family, close friends) and outsider-communication (wairen (外人) [65, 66]. Within insider-communication trust is high, so communication tends to be more direct and open. This contrasts with outsider-communication, when trust is low [65].

So in terms of Sihui’s discussion of her mother’s comments above, such interactions take place within high trust relationships in which honesty is prized. Living in a Chinese patriarchal society, the mothers want their daughters to succeed in a competitive context in which the currency of thinness—and certain standards of beauty—offer particular rewards and opportunities for young women. In this regard, there is a desire for their daughters to fit in and utilize their beauty and/or body to gain social benefits or economic success [47]. The fact that it was often mothers that emerged within these narratives is also not surprising. As women, mothers are themselves more at risk from gendered appearance judgement, and likely more aware of the social consequences of not conforming to beauty norms. Yet whilst perhaps emerging out of honesty and good intentions (and it is not the aim here to contribute to the historical demonization of mothers within in ED literature [22]), the participants clearly understood their mothers to play a role in the production of regulatory discourse on their bodies. The fact that this was understood as both entirely normative yet *also* harmful and damaging supports the wider feminist argument that EDs are inextricably intertwined with the everyday contexts of normative femininity [20, 21, 24].

#### Regulation of body/beauty ideals: experiences in US/UK

Several participants offered markedly different appraisals of the sociocultural context for body, beauty and gender ideals when they left China for the West. So in reflecting on her time in the UK, Bagel observed how:

[I]n China maybe people care more about their figure, so after coming [to the UK]… no one cares much about it. They think that even if you’re fat, you’re still beautiful and attractive. You just need to be confident. But in China, people might judge you … I think a change in environment is liberating for someone like me who just came from East Asia because the British have different aesthetic values compared to what we face in our environment …

Similarly, according to Julia:

I hope that China’s society can treat beauty in a diverse and varied way like Western countries do. Regardless of whether a girl is fat or thin, or whether she has a big or small chest or buttocks, she has her own unique characteristics, and beauty cannot be generalized. This person may have her own unique charm... They won’t say things like, ‘Why is this girl wearing a bikini on the beach? She’s such a slut’, or make comments about how she must be doing something improper … In Western countries… [t]hey appreciate your courage to wear whatever you want. But in China, if you post pictures of yourself in a bikini, you’re bound to receive … negative comments, as well as … ‘Aren’t you afraid your parents will see this?’

Both participants make reference to a perceived change in gender ideals here, whilst Julia extends this to consider the significance of broader gendered judgements—referencing perceptions of greater parental control over female agency and sexual autonomy in the Chinese context [15].

Notably, these comments contradict the contours of the traditional acculturation thesis in which (due to acculturative stress and the stronger influence of Western culture) ‘migrant’ women are at greater risk of developing EDs than their domestic peers (e.g. [67]). Quite the opposite discourse is relevant here: the participants found freedom in a Western ‘escape’ from their home context which they understood as strongly influencing their risk for EDs. As one participant pinpointed in seeking to explain the shift from East to West, the ‘judging environment has changed’ (Xiaoxi).

Other participants confirmed the perception of Western contexts as more liberating in the regulation of cultural body ideals. But the recognition of these contexts as essentially transient and temporary could also lead to *increased* self-surveillance and regulation:

I used to like [the clothing brands] Lolita and JK uniforms and most of my clothes were brought from China. Because those clothes had very strict size requirements, it might have *made me more conscious* about my weight [in the UK], as I had to control it so I wouldn’t outgrow the clothes [our emphasis] (Sihui).

Sihui talked of how it *was* easier to gain weight in the UK (changes in food; a more relaxed ‘judging’ environment), but as this was a temporary context, her original clothes acted as physical reminder of Chinese beauty standards to which she should still adhere. In this regard, even whilst the cultural environment was understood as less punitive in its prescription of bodily regulation, it led to greater personal conflict in terms of gender norms, weight and eating.

Either way, it may be difficult to align the participant perceptions here with existing discourse on body image in the West. Although health systems and governmental discourse clearly differ, both the UK and the US currently assert the existence of an ‘obesity crisis’ and fat shaming—which is historically gendered—is endemic, normalized and pervasive [68, 69]. Within this context, the comments from Bagel and Julia in particular (‘You just need to be confident’) perhaps resonate with what the UK scholars Gill and Orgad [70] have called the gendered address of ‘confidence culture’—a popular ‘feminist’ pushback against the gendered policing outlined above. As they explain, ‘love your body’ (LYB) discourse is a constituent part of confidence culture. Taking in multiple sites but often centralizing advertising discourse, LYB discourse responds to earlier feminist critiques of the beauty and media industries in propagating and policing female body ideals. As Gill and Orgad explain, the discourse is powerful because it promise[s] a ‘warm, positive, encouraging intervention into women’s relationship to their own embodied selves.. interrupt[ing] the… normalised hostile judgement and surveillance of women’s bodies in contemporary culture’ [70]. At the same time, they position confidence culture as compliant with the individualizing thrust of neoliberal feminism, only briefly acknowledging the ‘cultural injuries inflicted upon women in a patriarchal society’ [70] before they are to be overcome (often through consumerism itself).

As this reminds us, Western culture is *itself* mutable and changeable in ways which inflect how it is experienced and taken up. But although the comments above may in part resonate with Gill and Orgad’s paradigm, it has complex and uncertain implications for the participant responses. In the interview data in general, participants made very little reference to *any* kind of media discourse (Western or otherwise), and as in China, the experiences of the UK and the US put more stress on the importance of peer comments on body/beauty standards. Whilst these may circulate in the same discursive environment as (popular feminist) advertising rhetoric, they cannot be reduced to it. Gill and Orgad’s analysis is also part of a wider perspective on contemporary popular Western feminism which, whilst persuasive, often fails to take into account questions of reception or audience [71].

Indeed, if we look at the experiences of the women, it was clear that the felt experience of cultural difference could have *very real effects* on participant practices of eating and weight management:

[A]fter I came to the UK, I completely regained consciousness. I started to have regular meals, and I learned how to eat what I wanted... I think it might sound ridiculous to others, but I actually re-taught myself how to eat two years ago. You have to teach yourself everything again. I think it’s a very self-aware process (ChengCheng).

In pointing to the process of recovery and need to effectively ‘learn’ what is understood as more intuitive eating, ChengCheng presents the UK environment as a context in which she ‘regained consciousness’ and recognised the restrictive and damaging nature of her binging and purging. In a similar vein, Xiaoyang outlines the very real shift in her ‘self-identity’ that was fostered by her experience in the US:

[W]hen I went to the United States… nobody ever criticized me for being overweight… My confidence increased in all aspects …. I felt like I had reached the peak of my self-identity… No one had ever said anything bad about me … I really felt like even if I farted, someone would say it smells good (Xiaoyang).

Here, Xiaoyang presents the US as a kind of utopian, liminal space in which gendered judgements (the metaphorical use of the farting example) are somehow erased. This may be linked to her offering a rather rose-tinted view of a new country: being immersed within the everyday culture of a context is clearly different to visiting it. Xiaoyang indeed later acknowledged how ‘perhaps because I’m a foreigner, in Western culture people are more likely to praise your good points without mentioning any flaws’ (which offers an interesting inversion of the possible racism that may actually be experienced by Chinese students overseas) [72]. In this regard, she acknowledged that such an approach may have been indicative of an exaggerated ‘politeness’, offering a heightened discursive contrast with the rhetoric of ‘insider-communication’ discussed above in relation to the Chinese context.

Although both feminist and sociocultural perspectives recognise EDs as multifactorial in origin [73], these comments add credence to the view that social and cultural contexts play a *central* role in the development and experience of EDs. But the participant responses suggest how this process is deeply intertwined with discourses of cultural specificity in ways which may question or complicate existing paradigms—particularly in terms of Westernization. Although the geographical shift had different implications for different participants, the participants were responding to what they saw as a different set of cultural and communicative codes and discourses when it came to gendered cultural appraisals of their bodies and identities. Far from the West primarily representing an externally imposed pressure to conform to slimmer body ideals, experiences of gendered bodily regulation in China reframed experiences of Western contexts as ‘freeing’.

### Theme #2: discipline, appetite and control: the gendered/cultural meanings of binging and purging

The previous section focused on the regulation of body ideals and experiences of the perceived differences between China and the US/UK. But understanding eating problems as primarily issues of body image may over-emphasize the centrality of appearance to EDs and miss the complexity of gendered meanings imbricated within eating/body distress [9, 27]. In addition, AN has been consistently privileged within feminist and transcultural work, contributing to the cultural, medical and scholarly hierarchy in which AN and BN are often caught. When BN or BED are referred to in research on the Chinese context there is no discussion of the differences between EDs, or the cultural meanings of binging/purging (e.g. [54, 55, 74]). The data in our study suggests the vital importance of approaching ED problems other than AN through a transcultural lens.

The participants often understood EDs as being related to interpersonal strategies of control, thus marking similarities with Western scholarship and clinical discourse [9]. Either they suggested that the female body was so regulated externally that it emerged as a *central* focus of personal control, or that it was easier to control the domain of one’s own body rather than extraneous factors (gendered factors, educational or work stress). The currency of the second perspective is suggested by Xiaoyang in her recollection of how:

I saw a post in a newspaper … the girl who posted it wanted to study medicine, but …the educational requirements were high, and it was thought better for a girl to settle down early and be obedient. Her parents were also more traditional. Later on… she started to develop some binge-eating habits to alleviate her stress …. One reason is that it comes from the external demands on you… the moral demands on women are higher…And maybe eating [and] …. weight is one of the few things they can really control, and it also meets society's standards. Because as long as you cover up this thing like a lid, others can only see a pretty little girl who is quite thin… so sometimes I feel very sorry for them, you know?

This echoes de Montgrémier et al.’s study of EDs in young Chinese women in which ‘Western-influenced ideal[s] of independence’ conflict with more traditional expectations of filial piety, domesticity and obedience [15]. But although pointing to a similar gendered context, Xiaoyang’s response also asks us to consider the significance of binge-eating (and as she went on to explain, purging) within this environment. In suggesting that society can ‘only see a pretty little girl who is *quite* thin… [our emphasis]’, she lends credence to the argument that the binging/purging body is more inscrutable as a visual text [38]. Indeed, binging/purging was figured in the data as an example of how a woman can ‘explode in silence’ in China because she has little outlet to express such gendered oppressions (Penguin). As Penguin continued, the ‘binge eating of women… is related to the expectations of women's roles in East Asia’. Binging and purging were understood to be pervasive among young Chinese women, but also heavily stigmatized and silenced. As Amanda reflected: ‘I think [it is]… a very common phenomenon, but it's like an elephant in the room. And it's an invisible elephant. Everyone knows about it, but [they]… they won't easily poke at it’.

Within both Western medical and popular discourse, BN is often understood as representing a lack of control, whereas AN is figured as a form of over-control [29]. In terms of the gendering of this dualism, AN is constructed as signifying self-control, persistence, obedience, transcendence and purity, whilst BN is associated with chaos, moral weakness, sexual promiscuity and ‘mess’ [29]. Thus, even whilst AN is also pathologized in medical and cultural discourse, this opposition elevates self-starvation in ways which function to regulate understandings of desirable feminine norms. In the data for this study, AN was indeed more likely to be venerated than BN/BED. So as Penguin observed, ‘I think [it]… is mythologized… I searched online for how to get anorexia… [and] I would envy anorexic people’. The same association between self-control and morality also functioned to stigmatize the larger female body. As Ivy explained, if a girl is ‘overweight, we tend to think she lacks self-discipline’. What the women referred to as BN or BED (and as noted, cultural differences were at work here in terms of terminology), were also often spoken about in terms of a lack of control, but with the difference that the individual was ‘caught in a lie’ (Sihui). So in explaining why binging and purging were so shameful and ‘hidden’ within Chinese society, Sihui elaborated how:

I think one reason is that ... it is not the method of weight control that society shapes for us. If we accept the premise of the white, slim and young aesthetic, it would want you to be either genetically blessed, self-disciplined in exercise or eating very healthily to achieve this, but we achieve it through another method. It seems to imply a lack of self-control, that you have a problem as a person…. Many people would say that I couldn't control my mouth and was wasting food, which I think is a normal reaction (Sihui).

In the analysis below, we take different aspects of this quote in turn. First, Sihui refers to achieving the ideal body aesthetic through ‘self-discipline’. There was indeed a certain amount of emphasis on self-discipline, exercise and ‘clean eating’ in the data which could be considered in relation to debates about the rise of neoliberal healthism in China and its individualising challenge to collectivist discourse [75]. But this could equally be read in relation to the much longer historical emphasis on self-discipline as one of the cornerstones of Confucianism (pertaining to all aspects of cultural life from personal ethics, relationships, family, work or education [76]). In traditional Confucian thought, self-regulation is understood as central to the interaction between the person and the collective: it is not only about a sense of self-management, but also the cultivation of collective discipline, success and strength in which ‘one uses oneself as a standard to regulate one's conduct in interactions with others’ [76]. Although such values may have been challenged in the post-Maoist era and the subsequent rise of individualism and consumerism, they continue to exert an influence [77].

According to Sihui’s quote above, purging is transgressive in part because it *circumvents* self-discipline: it is framed as an undesirable short-cut to achieving that which should be worked for (and there was a real emphasis on letting family and significant others down through this secret behaviour). Another participant observed how, in ‘East Asian culture… we … emphasize that we must endure hardship in order to succeed’ (Xiaoyang). For others, to display a lack of self-discipline here was deeply connected with morality: so for Amanda, it transgressed standards for being a ‘successful person in society’ and made you a ‘shameless’ being.

Yet whilst this may pivot on particular cultural specificities surrounding morality and self-discipline, in approaching these discussions, it is also difficult not to think of the Cartesian dualism which has been so widely used in feminist work on eating problems [20, 21, 29]. Within this dualism, the mind is associated with ‘male’ rationality, whilst the ‘feminine’ body ‘is that which lacks control and threatens to “overtake” the mind— “it … overwhelms… erupts and disrupts” [20]. AN has been positioned as exemplifying the cultural power of this dualism with the thin/‘anorexic’ body ‘as *the* controlled body [original emphasis]’ [21], whilst BN figures as its debased opposite, the body in control of the mind [29]. It has been argued, however, that such mind–body dualism is not in fact applicable to (‘holist’) Chinese thinking [78], including related conceptualizations of EDs. As Lee argues for example:

[T]he Chinese cosmological notion of humanity does not recognize the Cartesian body-mind split that underlies both asceticism and the Western culture. In fact, any epistemology that decontextualizes the body is unlikely to be widely accepted. Instead, Chinese people amplify the body as a complex symbol system, and endorse somatization as a powerful metaphor for orchestrating the social response to illness [49].

This is a highly complex philosophical and conceptual debate which cannot be explored fully here. But in terms of wider philosophical work, it is worth noting that the idea of a sharp cultural contrast in this regard has been critiqued, and processes of cultural exchange explored [78, 79]. In looking for difference and specificity in the empirical, transcultural study of EDs, it is also crucial to be alive to similarity and continuity. So in discussing their binging and purging, the Chinese women in this study clearly spoke of a split between mind/body when discussing what they saw as their loss of control:

I could feel how full I was, I could feel that my stomach was about to burst, but I just couldn't stop. My brain couldn't make me stop… I think it completely dismantled me as a person, that is, it separated my mind and my body, and I no longer felt that they were one entity (ChengCheng).

Although ChengCheng’s reference to ‘I no longer felt they were one entity’ might refer precisely to a longer cultural heritage or perspective in which mind/body are *not* necessarily framed dualistically, the ED is clearly understood as ‘dismantling’ this framework. Much of the data in this regard was similar to the descriptions in Burns’ [29] Western qualitative study of womens’ experiences of binging and purging, marking out cultural similarity rather than difference.

But to return to a contextualization of the cultural meanings at work in binging and purging here, in Sihui’s original quote above, purging is also seen as unacceptable because it is understood as *wasting* food (‘people would say that I couldn't control my mouth and was wasting food’). In their qualitative study of the potential tensions between traditional eating norms in China and the modernizing influence of the thin, female ideal, Ng et al. [48] discuss how food refusal *and* wastage were interpreted as disrespectful for a range of reasons, including ungratefulness, disregard for more senior members of the family’s experience with food poverty in the Cultural Revolution, and disruption to expectations of filial piety ‘(“It is considered important for a ‘good’ child to eat well”)’ [48]. Indeed, there was certainly an emphasis in the data on vomiting as both shameful *and* wasteful in the familial context (even though not all participants lived with their family). But these discussions of how much to eat also raise the question of ‘appetite’.

In Chinese culture (as in other cultures), the concepts of sex and food are interlinked, and as Kang et al. observe, ‘many expressions describe food and sex in terms of each other, such as “a beauty to feast one’s eyes on” and “when the belly is full, the mind is among the maids”’ [80]. These quotes are clearly gendered, and apparently privilege a male desiring/eating subject and a female object (to be consumed). Yet little of the discussion of self-discipline, self-restraint and indeed appetite in relation to Chinese culture seems to consider these gendered differences. So when Farquhar notes how the ‘indulgence’ of personal appetites was more permissible after China’s transition from a Maoist culture (1950s to late 1970s) to one of post-socialism (with its greater emphasis on individualism and consumerism) [77], it seems pertinent to ask how such discourses are regulated by gender.

In the West, there is a long history of feminist work which examines the gendering of female appetite and food consumption which has at its core a ‘relationship between morality, sexual control and desire’ [68], and this has been taken up repeatedly in work on EDs [20, 21, 23]. Within this context, and despite the cultural polarisation of AN and BN, they are understood to represent inseparable ways of coping with cultural imperatives of slenderness and the greater regulation of female appetites [29]. As noted earlier, the participants in this study spoke little about sexuality and sexual relationships, and when they did, the context was always heterosexual. Some discussed how this functioned to regulate ‘appropriate’ constructions of female appetite:

When…. I started dating... I unconsciously began to persuade myself to behave like the kind of girl … [my boyfriends] expected me to be… When we went out to eat, I could eat a whole bowl of noodles, but I would only take a bite or two and say I was full… Once you start putting yourself in a heterosexual relationship, you start to convince yourself to reduce your appetite …. gradually conforming to the societal expectations of women (Penguin).

Others offered recollections of situations in which their appetite for food had been shamed. So QianQian recalled a comment from a work dinner when she took a second helping: ‘Aren't girls supposed to be thin? You … eat quite a lot’ (QianQian). Binging was articulated as a transgressive act (‘I can eat as much as I want without caring about what others might say’ (Julia)), whilst its privatised and ‘shameful’ nature spoke precisely to the gendered policing it was trying to avoid or subvert. Indeed, some of the participants shifted between acknowledging the subversive nature of female binging/purging (as ChengCheng eloquently put it, ‘a woman who cannot control her own process… has always been seen as dangerous in Chinese society’), and expressing extreme disgust, fear and self-judgement at their lack of self-restraint and control.

But there were differences across the interviews here, and not all described the relationship between gender, ‘everyday’ eating practices and EDs in this way. For some, the ideal woman in China who was someone who was seen to *eat well but remained thin*, offering an apparent contrast with histories in the West:

But in China, it seems not to be like that. If you are a person who really cannot eat anything, everyone will despise you… I feel that on the one hand, people think that girls should be thin and it looks good, but on the other hand, they seem to envy people who can eat a lot… it seems that Chinese people have a high pursuit of food and a high pursuit of thinness, and these two things are actually difficult to coexist… I feel conflicted about it. If you have no appetite, people will think you are not lively enough. But if you have an appetite and are also overweight, people will think you eat too much (Sihui).

For some participants, binging and purging emerged as an undesirable but understandable way to manage these contradictions—of relinquishing control over body and food, of respecting familial food cultures, and the need to display a female body which signified cultural imperatives of self-restraint (see also [48]). As Xiaoyang recalled for example:

I remember [an incident] when we …. we were eating Yoshinoya's double combo rice bowl. [My mum]… was eating too, but while putting her food in my bowl, she kept saying 'eat a little less.' I was very confused and asked her, 'Do you want me to eat less or finish my food? Why are you putting more food in my bowl and telling me to eat less?' … At first, many people are afraid you'll go hungry [but they also don’t want]… you to grow up as a girl who's too fat and bulky… So, at that time, I learned a little bit and figured out that I had to eat while hiding from them.

Struggling to negotiate conflicting imperatives around appetite (particularly within a familial context) and the stigmatization of (female) fat, the participant again relays how they ended up eating in secret, and alone.

Riebel observes how, in transgressing conceptions of femininity based on selflessness and conformity, the fear is that a binging woman ‘might consume what could literally feed a whole village’ [81]. Xiaoyang indeed went on to recall a vivid scenario in which she:

… would finish school and then head straight to the snack street …. and eat all the way through… I would engage in this kind of food scavenging behaviour … So, I would eat my way through every pawnshop on the street. I would eat my way back home, even though I still had to have dinner... I would eventually vomit not because I wanted to be skinny or beautiful, but simply because I had overeaten.

Xiaoyang describes how she ate her ‘way back home’, as if she is consuming her physical environment before reaching her destination. Interestingly, although the binging/purging body has been understood as a visceral production of a ‘profoundly lived body’ as opposed to the apparent denial of body suggested by constructions of AN [38], Xiaoyang later also drew attention to what she called ‘the joy of vomiting’—how she ‘really enjoyed the feeling of being filled up like a balloon and then flying away with a "pop”’. Here, binging and then purging is figured as way of *transcending* the body—a momentary release from the bodily processes of ingestion and expurgation and the unending wrestle with control.

## Conclusion

Over 50 years into the development of feminist scholarship on EDs, and decades into the enterprise of transcultural work in the field, more research is needed on how women from different cultural groups make meaning of body/eating distress. In drawing on 12 interviews with young women in urban China, this article has aimed to contribute to this critical project, examining how the participants understood the relationship between their own eating problems and cultural contexts of gendered power.

The first theme (‘Chinese versus Western codes for judging female appearance: from surveillance to liberation’) suggests the importance of examining contextually specific gender experiences of the interaction between Western and non-Western cultures—nuances which cannot be captured fully by metanarratives, quantitative measures or overriding assumptions about the ‘toxin’ of Westernization. Although Western ideals of (greater) freedom have shaped conceptions of women’s roles and oppression in ‘other’ cultures and countries [17], transcultural conceptions of EDs have often posited Westernization as an oppressive force which disrupts more ‘authentic’ and localized forms of embodiment and eating. But the data in the current study suggests that such comparisons are relative to lived cultural experiences and comparisons. Different communication codes and peer interactions across Chinese and Western contexts played a central role in how participants experienced the regulation of their bodies. This then had *real* impacts on eating habits, weight regulation and self-identity. Whilst the influence of Westernization may well have fostered a greater pursuit of the thin ideal in the Chinese context, this clearly interacts in distinctive ways with cultural specificity.

From the perspective of the participants, communication codes and contexts around gendered bodily regulation were understood as socially *normative* but also as harmful and triggering in relation to eating/body distress. Whilst clearly pivoting on culturally-specific experience, this supports the feminist argument that EDs are intimately imbricated within everyday and normative femininities. At the same time, although questioning the primacy of individual pathology may help to reduce self-blame, feminist paradigms have been accused of offering no easy solution for resistance or change if the experience of gendered cultural *environments* is so enmeshed with ED risk and aetiology [28]. After all, it is no simple thing to resist the power of social norms and mores and ‘remove’ yourself from your cultural context—as the participants studying in our sample had the privilege to do. Furthermore, despite the markedly different cultural experiences of China and the UK/US that were clearly understood as both positive and agentic for the participants, there is also something poignant about them seeking freedom and solace in countries where EDs are also pervasive, rife and deadly.

In terms of theme #2 (‘Discipline, appetite and control: the gendered/cultural meanings of binging and purging’) the article has emphasized the importance of examining the culturally specific meanings of eating problems which sit on the derogated side of an ED binary which privileges AN. In existing transcultural studies, the meanings and experiences of BN or BED are ignored or folded in with AN so that the centrality of thinness/starvation prevails. In offering what is only a start to approaching the meanings of meanings of binging and purging in the Chinese context, the data was multifaceted and complex. Binging and purging (in private) emerged as a way to manage a number of contradictions surrounding Chinese femininity, including respecting familial food cultures, contradictory discourses on female ‘appetite’, and the need to display a female body which signified cultural imperatives of self-restraint and discipline. But this invoked (and could be read in relation to) a range of contexts and discourses which relate to both Chinese and Western influences, and these are not simple to disentangle.

Indeed, in terms of how the women spoke about the gender ideologies and inequalities that structured their experience of their bodies and identities within a patriarchal culture, there is much that echoes Western feminist work on Western samples [21, 28, 29]. Although culturally-specific structures and experiences of embodiment, appetite, food and gender roles are crucial, ‘the oppression of women and the maintenance of patriarchal control, are constants found across many different cultures…’ [33]. So in their earlier article, Katzman and Lee suggested that ‘no control phobia’, as opposed to ‘fat phobia’, was a more useful transcultural feminist discourse for understanding EDs [9]. But a key point of their intervention was to suggest the ways in which transcultural studies could and should be providing empirical evidence to feed back into the parameters and epistemologies of feminist paradigms, strengthening their interventions into conceptual, theoretical and clinical understandings of eating problems. One of the greatest strengths of the feminist approaches has arguably been the use of empirical data—listening to the voices of those with experience. Huge scope remains for transcultural, feminist studies to occupy a more central space in this dialogue.

### Limitations

A significant proportion of the sample occupied a sexual minority identity, but the study did not yield significant data on the implications of this for the aetiology or experience of an ED. Based on existing qualitative research which details how experiences of sexual marginalization can shape the meanings of eating/body distress [36], this may be a rich terrain for further transcultural research in China. The sample in the study is obviously small, and it includes a particular group of well-educated women from urban areas in China. Greater variability in region and educational background could yield different results. Indeed, many of the participants were already accustomed to understanding and critiquing the world in gendered terms in ways which made them articulate and forthcoming subjects for this study. Although there have been signs of change within popular discourse, the concept of feminism in China remains deeply contested, ambivalent and stigmatised within everyday life [82]. Whilst we did not ask the participants if they identified as ‘feminist’, it would be interesting and revealing to explore how participants without similar educational backgrounds discussed the relationships between gender and EDs in this context.

If a combination of feminist and transcultural paradigms appear useful for approaching and understanding EDs in the Chinese context, then this has clear implications for treatment [15, 33]. Some of the feminist approaches to EDs originally emerged out of clinical practice [18, 19], but for a range of epistemological, institutional and cultural reasons, they have since found themselves marginalised within clinical contexts (in the West and elsewhere) [28, 73]. Thinking about the transcultural relevance of feminist approaches in the Chinese context would be complex, requiring engagement with the epistemological status of feminist approaches in relation to ED treatment; specificities of the Chinese clinical context; the transcultural implications of these feminist approaches and (as part of this), Chinese engagement with concept of ‘feminism’ itself.

Feminist work has been criticized for its limited efforts to engage girls/women in *discussion and evaluation* of the feminist paradigms and their implications for understanding the development, experience and treatment of eating problems [28]. Given that, as with EDs, feminism(s) respond to, engage with and resist experiences of patriarchal power in both globalised and locally-specific ways, transcultural studies which engage participants in this evaluation could be an important place to start.

## Data Availability

The datasets generated and/or analysed during the current study are not publicly available due the parameters of consent obtained from participants.

## Abbreviations

EDs: Eating disorders
AN: Anorexia Nervosa
BN: Bulimia Nervosa
BED: Binge eating disorder
